# Rethinking Education in a Crisis: How New Is a New Common Really?

**DOI:** 10.1007/978-3-030-65355-2_20

**Published:** 2021-03-20

**Authors:** Max Louwerse, Marie Postma, Maarten Horden, Anton Sluijtman

**Affiliations:** 1 Tilburg University, Tilburg, The Netherlands; 2 Tilburg University, Tilburg, The Netherlands; 3 Tilburg University, Tilburg, The Netherlands; 4 Tilburg University, Tilburg, The Netherlands; 5 Department of Cognitive Science and Artificial Intelligence, Tilburg School of Humanities and Digital Science, Tilburg, The Netherlands; 6 DAF Technology Lab, Tilburg University, Tilburg, The Netherlands

## Abstract

The COVID-19 pandemic has disrupted the status quo in many areas of society, including education. At all educational levels, on-site lecturing had to switch instantaneously to an online mode of instruction. This transition was so straightforward, that the argument could be made for online education to become a permanent fixture, particularly if it is more efficient, cheaper, and more effective than traditional education. Extensive meta-analyses, however, show that most online teaching practices do not lead to better educational outcomes than the on-site alternatives. Worse yet, the traditional face-to-face mode of lecturing is ineffective in the absence of personalized interactions. The proposed solutions are offered by artificial intelligence research, including virtual reality, intelligent tutoring systems, and serious games—solutions that have so far not been extensively implemented in practice. The current health crisis provides our educational professionals with an opportunity to rethink their teaching practices and focus on applying these promising new alternatives.

The COVID-19 pandemic has disrupted the status quo in many areas of society, including education. At all educational levels, on-site lecturing had to switch instantaneously to an online mode of instruction. This transition was so straightforward, that the argument could be made for online education to become a permanent fixture, particularly if it is more efficient, cheaper, and more effective than traditional education. Extensive meta-analyses, however, show that most online teaching practices do not lead to better educational outcomes than the on-site alternatives. Worse yet, the traditional face-to-face mode of lecturing is ineffective in the absence of personalized interactions. The proposed solutions are offered by artificial intelligence research, including virtual reality, intelligent tutoring systems, and serious games—solutions that have so far not been extensively implemented in practice. The current health crisis provides our educational professionals with an opportunity to rethink their teaching practices and focus on applying these promising new alternatives.

In the months during which the COVID-19 pandemic dramatically affected the lives of many around the globe, society at large tried to find ways to adapt to the new circumstances. One sector of society, i.e., education, transformed almost instantaneously. From 1 day to the next, the usually vibrant sites of primary, secondary, and higher education emptied, and all educational activities switched to online. While the shift to online education has been challenging in many ways, the fact that the change could be effectuated so rapidly and, in general, quite successfully raises the inevitable question whether online education should not become an integral part of the way children, students, and adults receive formal education—a new common.

One of the very few benefits of a crisis like COVID-19 is that it forces society to rethink aspects and processes that are traditionally resistant to change. Education is one such aspect. Due to the pandemic, millions of students had to be taught differently than before, and the disruption yielded the question what education actually entails. If lectures in which information is transferred can easily be offered online, is there any advantage to being an audience in a lecture hall compared to being an audience in front of a computer screen? If the difference is negligible, then the practical advantages of the new (digital) reality may outweigh any disadvantages. For example, online courses can be recorded once and used again, thereby freeing time for tailored lecturer–student interactions. Remote contact may feel less personal, yet, for the same reason, contact may be established easier by students who otherwise feel too timid to approach the lecturer after class. Since students do not need to travel to attend a lecture, participation barriers are removed for students residing at a distance from the campus. Given these advantages of online education, should online education become the new common, and if so, what is the best online education?

## The “Old” Common

Universally, physical lectures are by far the most commonly used form of knowledge transfer. As Bligh (1998) points out, despite the many research findings in educational psychology disputing the traditional teaching format and the developments in educational technology, there has been little change in the way people are taught around the globe. This is true not only for children but also for adults in the context of life-long learning. In fact, as demonstrated by old Roman reliefs and paintings originating in the Middle Ages throughout human history, becoming educated tended to be synonymous with being lectured. Despite Enlightenment and the Industrial Revolution, and with the digital society rapidly changing our interactions with the world and each other, the status quo of education has been remarkably resistant to change. This would be understandable if the common form of being educated were superior to any of its alternatives. That, however, is hardly the case.

Based on an extensive review of educational practices, Bligh (1998) concludes that lectures offer little promise in inspiring thought or attitude changes. In another extensive meta-study, Hattie (2015) evaluated some 1200 peer-reviewed meta-analyses on student achievement and reported 256 variables that affected student achievement positively or negatively. Unsurprisingly, student-dependent variables were the greatest source of variance in learning, and variables such as ADHD, deafness, and depression affected achievement negatively. The second most important source of variation, however, were teacher-dependent variables. As Hattie noted, most educators were themselves successful as students in the classical classroom setting and may thus belief that the setting provides optimal conditions for learning if the students apply themselves. Yet, it is the educators’ awareness of how to achieve impact in a teaching situation and their willingness to change and adapt that are the most important contributing factors to the learning success of their students. According to Hattie, the question we need to ask ourselves is not “What works?” but, rather, “What works best?”

The conclusion Hattie (2015) draws is echoed in the meta-analysis of Schneider and Preckel (2017). They observed that most teaching practices may give rise to positive effect sizes with regards to learning achievement, but some have considerably larger effect sizes than others. In other words, if we are striving for excellence, not every teaching method goes. In practice, evaluating what works best may be difficult as it depends on the comparisons being made as well as on the type of education being looked at. For instance, universities are more selective than primary or high schools, so it is likely that the average university student population consists of highly motivated individuals. University students also have more experience with classical formal education. According to Schneider and Preckel’s analysis, however, also for these students, there is a clear advantage of tailored interaction. Encouraging frequent class participation, stimulating questions and discussion, and asking open-ended questions tend to improve the instructional quality of courses. Ironically, the ambition to provide high-quality university education to ever-growing numbers of students has resulted in overcrowded lecture halls and a dramatic decrease in the opportunity to interact and to stimulate questions and discussion, thus rendering the traditional mode of knowledge transfer ineffective.

## The “New” Common

When the COVID-19 pandemic hit societies across the world, on-site lectures transformed into online interactions. The transformation was necessary and seemed to work well given the unprecedented circumstances. According to Schneider and Preckel (2017), online education in general seems to be a decent substitute for on-site education. Online learning turns out to be almost as effective as learning in the classroom. If student achievement in online education is on par with on-site education, it would be wise to invest in online lectures and podcasts, massive open online courses (MOOCs), and online learning platforms not only during a COVID-19 crisis but also beyond.

However, studies focusing on the comparison between online and face-to-face courses come to a different conclusion. Xu and Jaggars (2014) analyzed the data from half a million courses taken by over 40,000 community and technical college students and found lower overall grades in the online sections of the same course compared to the face-to-face sections. Moreover, males, younger students, black students, and students with lower grade point averages yielded the strongest declines, suggesting a performance gap between online and face-to-face modes of interaction. An extensive analysis of MOOCs by Reich and Ruipérez-Valiente (2019) used the data from MIT and Harvard MOOCs, including 565 course iterations from 261 different courses, with 12.67 million registrations. The dropout rate was 96% on average over a period of 5 years. Of course, it can be argued that completion rates are not the goal. It may be the case that enrolled students are merely curious and then drop out. That in itself would be an interesting finding, but Reich and Ruipérez-Valiente (2019) noted that course completion remained relatively low even among students who pay for courses, and, interestingly, they observed similar differences across types of students as Xu and Jaggars (2014). Arguably, the negative effects of online teaching could partly be alleviated if online education is combined with on-site education in blended learning, but this step would only be successful if we can avoid the problem of large classrooms and little personalized student–lecturer interaction in the first place.

## AI in Education

If there were no alternatives to the two scenarios described above, we might be justified in concluding that the traditional mode of lecturing, while not ideal, is the best we can do. Yet the alternatives are there, and their effects on student achievement are promising. In particular, games in virtual reality and interactive virtual reality simulations of real-world processes have a positive impact on student performance that is considerably higher than online education (Hattie 2015; Schneider and Preckel 2017). A meta-analysis of studies on the effectiveness of virtual reality-based instruction on students’ learning outcomes in K-12 and higher education showed that games, simulations, and virtual worlds were all effective in improving learning gains. Even though it is true that these technologies involve starting costs in software development, computer hardware, and instructional time, the scalability of the solutions is likely to outweigh the investments needed.

What could a new common in education consist of? Louwerse et al. (2020) argue that immersive education could consist of virtual reality in which the content of education is brought closer to the learner and vice versa. It could consist of serious games that allow for bringing together excitement and learning, for instance by exploring past times through the (virtual) eyes of those who lived during those times. It could consist of intelligent tutoring systems that provide personalized education 24/7, for instance by a computer having a conversation with a student in natural language (Graesser et al. 2004). It could consist of utilizing learner analytics, by measuring student progress, not after the educational process but during the process itself, intervening when intervention is most needed.

Tilburg University has been involved in a number of projects that investigate innovative educational technologies. For instance, on campus, the DAF Technology Lab offers a Cave Automatic Virtual Environment (CAVE) system that provides immersive education to students. In one of the experiments we conducted, we investigated learning gains in a neuroscience class presented in virtual reality, whereby students were asked in the CAVE system to link brain structures to their labels, their functions, and the brain locations. The findings demonstrated that students yielded higher learning gains in the virtual reality environment than in traditional educational settings, such as textbook learning. This experiment was conducted using both a college subject pool population and a group of students in a cognitive science course that involved a neuroscience class (de Back et al. 2020). In addition, the research in the DAF Technology Lab has shown how to measure neurophysiological data that cue learning in students (Tinga et al. 2019).

In another project, we collaborate with the non-profit organization SpaceBuzz to create ambassadors of planet Earth. The result of the collaboration is an innovative educational program aimed at introducing primary school education to the subjects of science and technology in the context of sustainability in a way that is playful and easy to learn. The program has been developed in line with the career path of a real astronaut. It consists of a pre-flight astronaut training, involving a variety of activities and lessons in the classroom that prepare children for their journey into space. After the children pass the pre-flight astronaut training, a 15-m long rocket arrives at the school to virtually launch the children into orbit. When the children sit down in the rocket and put the virtual reality headsets on, their chairs move hydraulically and the rocket is launched into space under the guidance of a virtual reality embodiment of an actual astronaut. Finally, in a post-flight training at the children’s school, the children give press conferences to friends and family and tell them about their experiences in space. An experiment with some 200 children from elementary schools has shown that learning gains ensued from the SpaceBuzz virtual reality experience and could be predicted through neurophysiology, specifically eye gaze (van Limpt-Broers et al. 2020).

## Conclusion

The COVID-19 pandemic has hit all aspects of society, including education. It has also provided us with an opportunity to rethink education, if not during the crisis itself, at least beyond the crisis. Extensive analyses in the educational psychology literature have shown that traditional forms of education are only effective if combined with small-scale, interactive, and tailored modes of instruction that are hardly achievable in the current educational landscape. Online education as such does not provide a viable alternative. In our view, the solution lies in the implementation of innovative solutions, such as those developed by our team at Tilburg University, in the DAF Technology Lab, and by SpaceBuzz. These educational solutions offer exciting new avenues of investigating what a new common really should consist of.
